# Quantum algorithm for MMNG-based DBSCAN

**DOI:** 10.1038/s41598-021-95156-7

**Published:** 2021-07-30

**Authors:** Xuming Xie, Longzhen Duan, Taorong Qiu, Junru Li

**Affiliations:** 1 School of Information Engineering, Nanchang University, Nanchang, 330031 People’s Republic of China; 2 School of Software and Internet of Things Engineering, Jiangxi University of Finance and Economics, Nanchang, 330013 People’s Republic of China

**Keywords:** Computer science, Information theory and computation

## Abstract

DBSCAN is a famous density-based clustering algorithm that can discover clusters with arbitrary shapes without the minimal requirements of domain knowledge to determine the input parameters. However, DBSCAN is not suitable for databases with different local-density clusters and is also a very time-consuming clustering algorithm. In this paper, we present a quantum mutual *MinPts*-nearest neighbor graph (MMNG)-based DBSCAN algorithm. The proposed algorithm performs better on databases with different local-density clusters. Furthermore, the proposed algorithm has a dramatic increase in speed compared to its classic counterpart.

## Introduction

Clustering, an important branch of unsupervised machine learning, is the process of partitioning a dataset into subsets of points called clusters, such that similar points are grouped in the same cluster and dissimilar points are put in different clusters. This procedure is widely used in many scientific fields, including bioinformatics^1–4^, image processing^5–8^, and social networks^9,10^.

DBSCAN^11^, a density-based clustering algorithm, is one of the most famous clustering algorithms. The distinguishing advantage of the DBSCAN algorithm is that it can be used to discover arbitrarily shaped clusters. Furthermore, it does not need the minimal requirements of domain knowledge to determine the input parameters, and can also exclude outliers from the clusters. However, DBSCAN has two dire drawbacks. First, DBSCAN has a low efficiency on databases with different local-density clusters; second, the algorithm is very time-consuming.

Quantum computing has attracted tremendous attention due to its parallel capability. In 1982, Feynman pointed out that quantum computers might achieve significant increase in speed over classical computers on certain specific problems^12^. Shor’s algorithm^13^ and Grover’s algorithm^14^ are two of the most popular quantum algorithms. Shor’s algorithm has an exponential increase in speed, and Grover’s algorithm has a quadratic increase in speed over their classical counterparts. With the rise of quantum computing, many researchers have also designed various quantum machine learning algorithms and quantum data mining algorithms, such as quantum linear regression^15–17^, quantum support vector machine^18,19^, quantum k-nearest neighbors classification^20^, quantum deep learning^21^, and quantum association rules mining^22^. Recently, tremendous advances have been made in constructing quantum computers. Krantz et al.^23,24^ introduced the central concepts and challenges of superconducting quantum circuits. Huang et al.^25^ provided experimental efforts toward large-scale superconducting quantum computers. Bruzewicz et al.^26^ concluded the basics of trapped-ion quantum computing and explored the outlook for trapped-ion quantum computing.

Inspired by quantum computing, we propose a quantum mutual $MinPts$-nearest neighbor graph (MMNG)-based DBSCAN algorithm. First, we design a quantum mutual $MinPts$-nearest neighbor graph algorithm that is devoted to dividing a database into subsets. After that, we quantize the original DBSCAN algorithm to cluster each subset.

## Preliminaries

In this section, we provide the necessary background knowledge for this paper. First, we briefly introduce the basic definitions of the classical DBSCAN algorithm. Then, we review the fundamental concepts of Grover’s algorithm.

### DBSCAN

The DBSCAN algorithm offers a new notion of “cluster” and “noise” in a database $D$ of $N$ points of some k-dimensional space $S$. The whole set of definitions is given as follows.

#### Definition 1

($Eps$-*neighborhood of a point*) The $Eps$-neighborhood of a point $p$, denoted by $N_{Eps} (p)$, is defined by $N_{Eps} (p) = \{ q \in D|Dist(p,q) \le Eps\}$.

The $Eps$-neighborhood, the fundamental definition of the algorithm, can be used to distinguish core points and noncore points. $Eps$ is the distance threshold. Core points are the points inside of any cluster, and noncore points are the points on the border of any cluster or the points belonging to none of the clusters. Let $p$ be a point in a database $D$, where $|N_{Eps} (p)|$ denotes the number of points within the $Eps$-neighborhood of $p$. Let $MinPts$ be the threshold of the number of points; if $|N_{Eps} (p)| \ge MinPts$, then $p$ is a core point; otherwise, $p$ is a noncore point.

#### Definition 2

(*directly density-reachable*) A point $p$ is *directly density-reachable* from a point $q$ if

1. $$p \in N_{Eps} (q)$$ and
2. $$|N_{Eps} (p)| \ge MinPts$$

Directly density-reachable is not always symmetric. When $p$ and $q$ are both core points, the direct density reachability is symmetric; when one is a core point and the other is a border point, the direct density reachability is asymmetric.

#### Definition 3

(*density-reachable*) A point $p$ is density-reachable from a point $q$ if there is a chain of points $p_{1} ,p_{2} , \ldots ,p_{N} \in D$, $p_{1} = q$, $p_{N} = p$ such that $p_{i + 1}$ is directly density-reachable from $p_{i}$.

#### Definition 4

(*density-connected*) A point $p$ is density-connected to a point $q$ if there is a point $o$ such that $p$ and $q$ are density-reachable from $o$.

#### Definition 5

(*cluster*) Let $D$ be a database of points. A cluster $C$ is a nonempty subset of $D$ satisfying the following conditions:

1. $\forall p,q$: if $p \in C$ and $q$ is density-reachable from $p$ then $q \in C$.
2. $\forall p,q \in C$: $p$ is density-connected to $q$.

In the database $D$, not all the points belong to clusters. The points that do not belong to any cluster are defined as “noise” in the DBSCAN algorithm.

#### Definition 6

(*noise*) Let $C_{1} , \ldots ,C_{k}$ be the clusters of the database $D$, $i = 1, \ldots ,k$. Then, we define the noise as the set of points in the database $D$ not belonging to any cluster $C_{i}$, i.e., noise = $\{ p \in D|\forall i:p \notin C_{i} \}$.

### Grover’s algorithm

Let us assume that we wish to search $M$($1 \le M \le N$) solutions from an unstructured search space of $N$ elements. Rather than examining $N$ elements one by one, Grover’s algorithm checks the elements in parallel by assigning indexes to all of the elements and storing the indexes in a quantum register. With a series of unitary operations augmenting the success probability gradually, Grover’s algorithm can obtain the indexes of the target elements with a high probability.

## The proposed algorithm

In this section, we design a quantum mutual $MinPts$-nearest neighbor graph algorithm and a quantum DBSCAN algorithm and present a quantum MMNG-based DBSCAN algorithm.

### Quantum MMNG algorithm

Let $D$ be a database, $p$ and $q$ be some objects in $D$, and $MinPts$ be a positive integer. The relative concept will be introduced as follow.

#### Definition 7

(*Mutual* $MinPts$-*nearest neighbor (MMN*)): If $p$ is in the $MinPts$-nearest neighborhood of $q$ and $q$ is in the $MinPts$-nearest neighborhood of $p$, then we call $p$ a mutual $MinPts$-nearest neighbor of $q$; similarly, $q$ is a mutual $MinPts$-nearest neighbor of $p$.

#### Definition 8

(*Mutual* $MinPts$-*nearest neighbor graph (MMNG)*): The mutual $MinPts$-nearest neighbor graph can be constructed by connecting each point to its mutual $MinPts$-nearest neighbors.

Note that MMNG is an algorithm with high complexity. To speed up the MMNG, we intend to quantize the MMNG algorithm. Dürr et al.^27^ developed a *quant_find_smallest_values* algorithm for finding the $c$ closest neighbors of a point with high probability within $O\sqrt {cn}$ time. Based on the *quant_find_smallest_values*, we propose a quantized MMNG algorithm, as shown in algorithm 1.
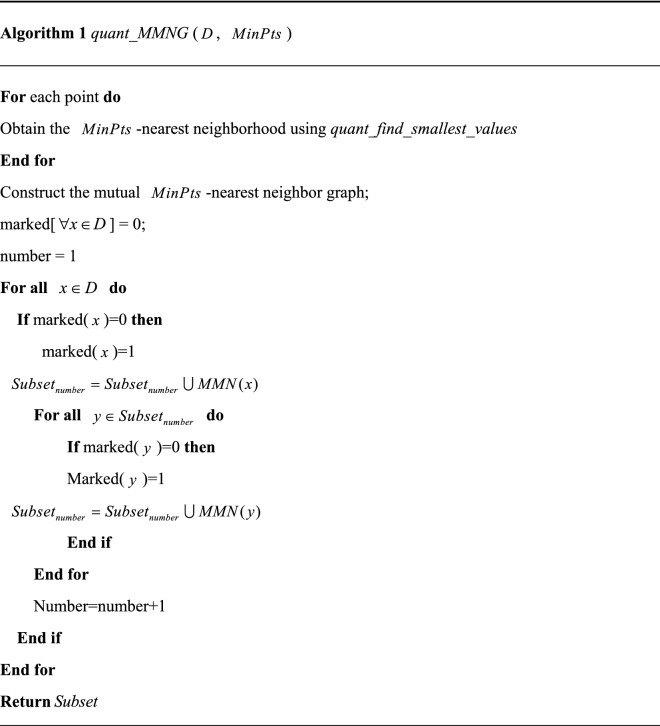


In this paper, algorithm 1 is used to obtain the subsets of database $D$. After obtaining the subsets, we apply the quantum DBSCAN algorithm on each subset to obtain the eventual clusters and the noise set.

### Quantum DBSCAN algorithm

We consider a database $D_{N} = \{ p_{1} , \ldots ,p_{N} \}$, which is composed of $n$ points, and each point $p_{i}$ has $k$ attributes. For each point $p_{i}$ in $D_{N} = \{ p_{1} , \ldots ,p_{N} \}$, it is necessary to calculate $Dist(p_{i} ,p_{j} )$ $n - 1$ times to determine the $Eps$-neighborhood of $p_{i}$. Determining the $Eps$-neighborhood is fairly time-consuming. To solve this problem, we intend to screen the points in the $Eps$-neighborhood of $p_{i}$ with quantum search.

**Figure 1:**
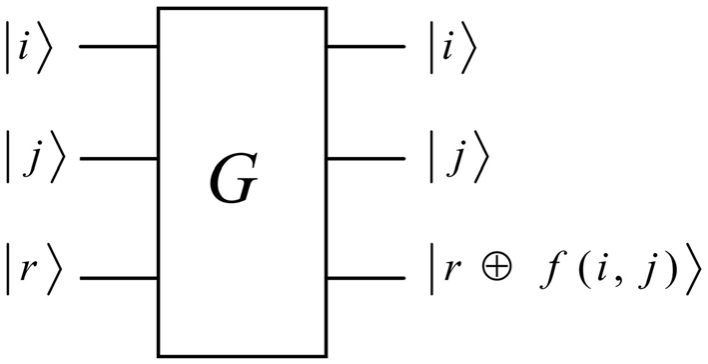
The oracle to compute the distance between $p_{i}$ and all the possible $p_{j}$s. $\left| r \right\rangle$ is an ancillary register.

In our model, a quantum distance black box is proposed. The proposed black box can accept two types of inputs, as illustrated in Fig. 1. $\left| i \right\rangle$ is a one-state input and the index of point $p_{i}$; $\left| j \right\rangle$ is a superposition of inputs and includes the indexes of all the points. Evidently, this is feasible because one q-bit can be a pure state or a superposition of states. Furthermore, one query to this black box means asking for distances between the point $p_{i}$ and all the points $p_{j}$ s (when $i = j$, $Dist(p_{i} ,p_{j} ) = 0$). After obtaining the distances, the black box compares them with the $Eps$ distance. Then, a selection function $f(i,j)$ assigns a value of 1 when $Dist(p_{i} ,p_{j} )$ is smaller than or equal to the $Eps$ distance and a value of 0 otherwise. The selection function is shown in Eq. (1).

1 $$ f(i,j) = \left\{ {\begin{array}{*{20}l} {\begin{array}{*{20}c} {0,} & {{\text{if}}} & {Dist(p_{i} ,p_{j} ) > Eps} \\ \end{array} } \hfill \\ {\begin{array}{*{20}c} {1,} & {{\text{if}}} & {Dist(p_{i} ,p_{j} ) \le Eps} \\ \end{array} } \hfill \\ \end{array} } \right. $$

Meanwhile, a flipping operation is carried out in the black box. As depicted in Eq. (2), if $f(i,j) = 1$, the ancillary register $\left| r \right\rangle$ is flipped; if $f(i,j) = 0$, the ancillary register $|r\rangle$ remained unaltered. The symbol $\oplus$ denotes module 2, also known as an exclusive-or.

2 $$ \left| i \right\rangle \left| j \right\rangle \left| r \right\rangle \to \left| i \right\rangle \left| j \right\rangle \left| {r \oplus f(i,j)} \right\rangle $$

Based on the aforementioned black box, we designed algorithm 2 (*quant_find_*$Eps$-*neighborhood* as described below) as a subroutine of the quantum-based DBSCAN algorithm. Given a specific point $p_{i}$, algorithm 2 is able to fix its $Eps$-neighborhood.
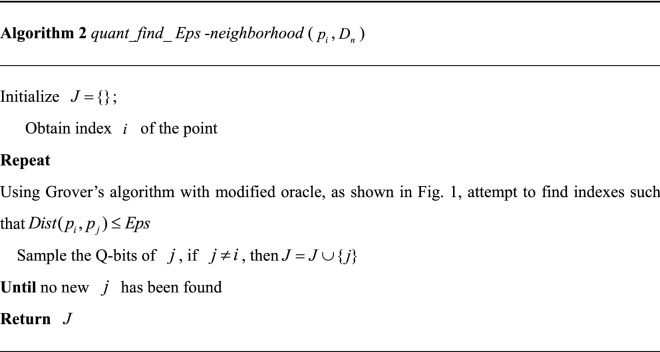


Once the $Eps$-neighborhoods are fixable quantum-mechanically, core points and noncore points become discernable according to the basic notions of the classic DBSCAN. If a point is a noncore point, we keep looking for a core point because there is no need to create a new cluster for a noncore point; however, if a point is a core point, we set up a new cluster and expand it. With the expanding methodology offered in the original DBSCAN algorithm, the quantum-based DBSCAN algorithm *quant_DBSCAN*($D_{N}$, $Eps$, $MinPts$) is presented hereafter, as shown in algorithm 3.
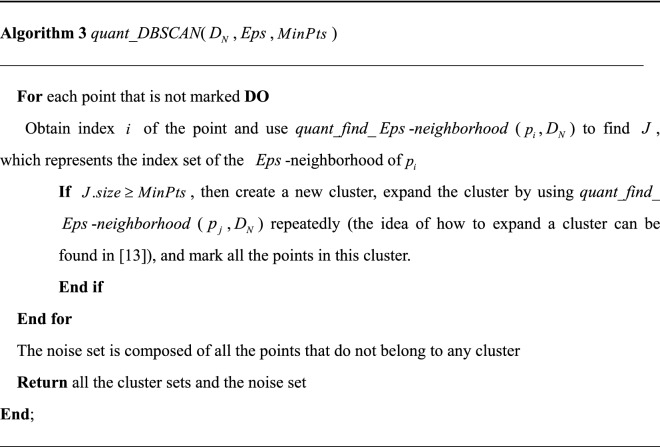


### Quantum algorithm for MMNG-based DBSCAN

The proposed algorithm divides the database into subsets first and then applies the quantum DBSCAN algorithm to each subset. Note that different subsets have a different $Eps$ in our algorithm. For a specific subset, we select the average $MinPts$ distance as the $Eps$ of the subset.
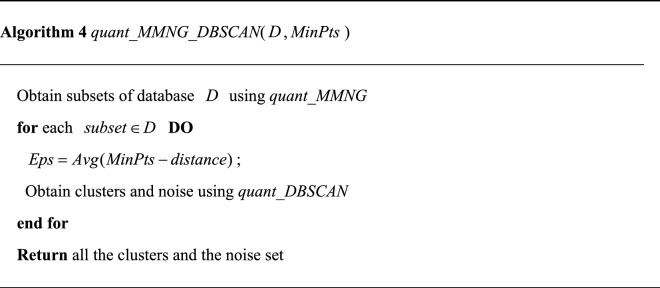


## The algorithm analysis

In this section, we briefly analyze the complexity of our algorithm first and then present the success probability of our algorithm.

### The complexity

Dürr et al.^27^ proved that the complexity of *quant_find_smallest_values* is $O\sqrt {cn}$. It is easy to see that the complexity of algorithm 1 is $O(N\sqrt {MinPts*n} )$.

For algorithm 2, there are $|N_{Eps} (p)| + 1$ targets for each point. According to the original version of Grover’s algorithm, algorithm 2 needs to interrogate the oracle approximately $\sqrt {\frac{N}{{|N_{Eps} (p)| + 1}}}$ times. It can easily be perceived that the smaller $|N_{Eps} (p)|$ is, the more queries are needed. In the worst case scenario, when $|N_{Eps} (p)| = 0$, the queries of the oracle are approximately $\sqrt N$ times. In other words, the complexity of algorithm 2 is $O(\sqrt N )$.

For algorithm 3, we need to calculate the $Eps$-neighborhood of every point. This means that algorithm 3 needs to call algorithm 2 $N$ times. Thus, we can ensure that the complexity of algorithm 3 is smaller than $O(N\sqrt N )$, even though the $|N_{Eps} (p)|$s are different for different points.

In other words, the complexity of our proposed algorithm is approximately $O(N\sqrt {MinPts*n} )$.

### The success probability

Dürr et al.^27^ proved that *quant_find_smallest_values* is able to obtain $c$ nearest neighbors with a high probability. It is easy to infer that algorithm 1 can obtain subsets with a high probability.

It is noteworthy that $|N_{Eps} (p)|$ s are different from point to point, which means that there are different numbers of targets when algorithm 2 is dealing with different points. As a result, the success probabilities are different when calculating different $Eps$-neighborhoods. By referencing the former work, the success probability of algorithm 2 can be calculated via Eq. (3) after $T$ iterations.

3 $$ P = \sin^{2} ((2T + 1)\arcsin \sqrt {\frac{{|N_{Eps} (p)| + 1}}{N}} ) $$

We already know that $T \approx \sqrt {\frac{N}{{|N_{Eps} (p)| + 1}}}$. Usually, $MinPts$ is far less than $N$, and $|N_{Eps} (p)|$ are numbers close to $MinPts$. As a result, it can be inferred that the success probability of algorithm 3 is high.

Our proposed method is a combination of algorithm 1 and algorithm 3, thus success probability of the proposed method is high.

## Performance evaluation

**Figure 2:**
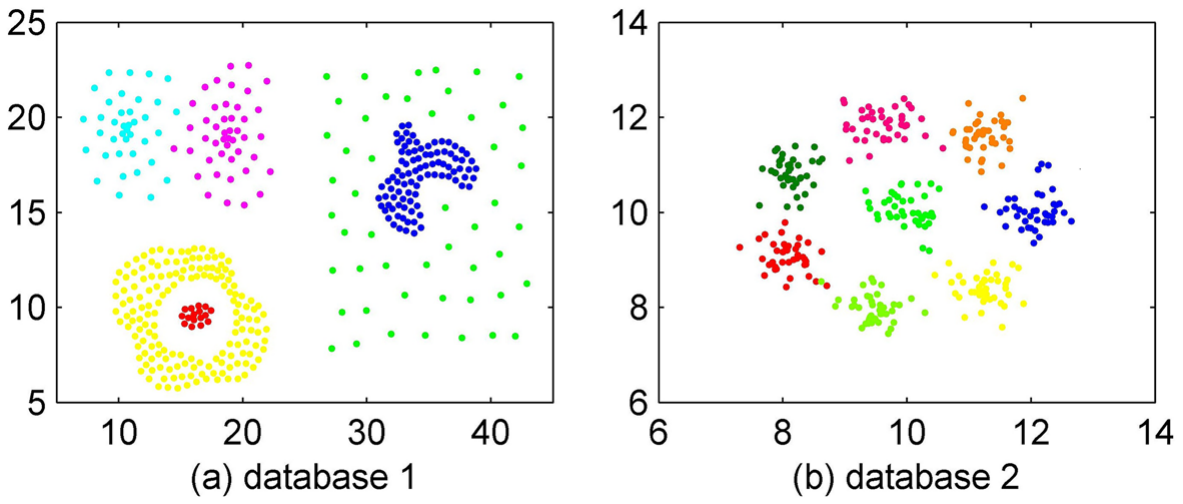
Sample databases.

To show the effectiveness of the proposed algorithm, performance evaluation based on two databases is conducted. To compare our algorithm with the classic DBSCAN method and the NaNG method, we use the two synthetic sample databases depicted in Fig. 2.

**Figure 3:**
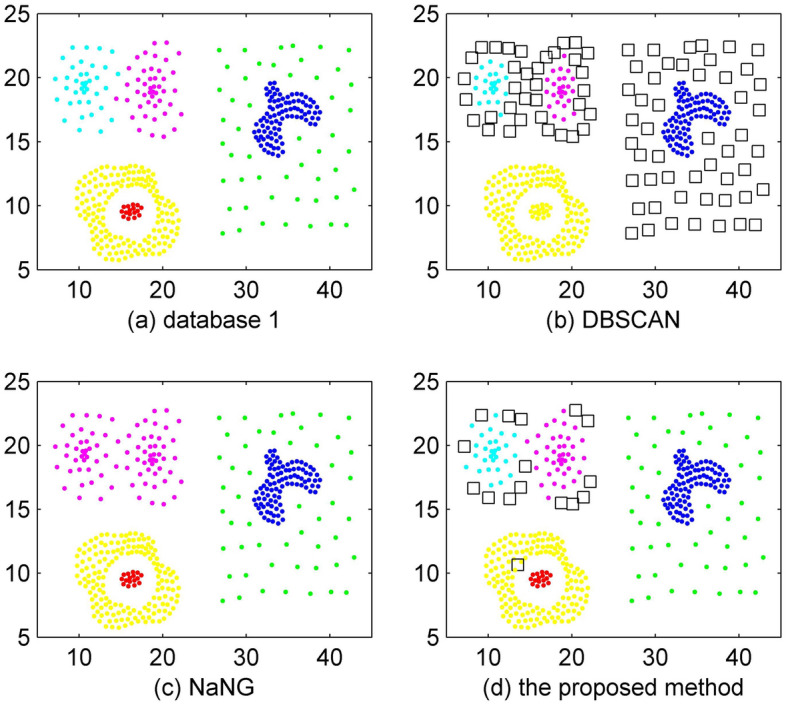
Clustering results of database 1.

The experimental results on database 1 are shown in Fig. 3. A total of 399 objects are included in database 1. In the figure, the black squares represent the points that are detected as outliers. The experimental result of DBSCAN on database 1 is undesirable, and the accuracy is approximately 74.6%. The experimental result of NaNG is better than that of DBSCAN, with an accuracy of 90.73%. Our proposed method has the best performance on database 1, with an accuracy of approximately 95.74%.

**Figure 4:**
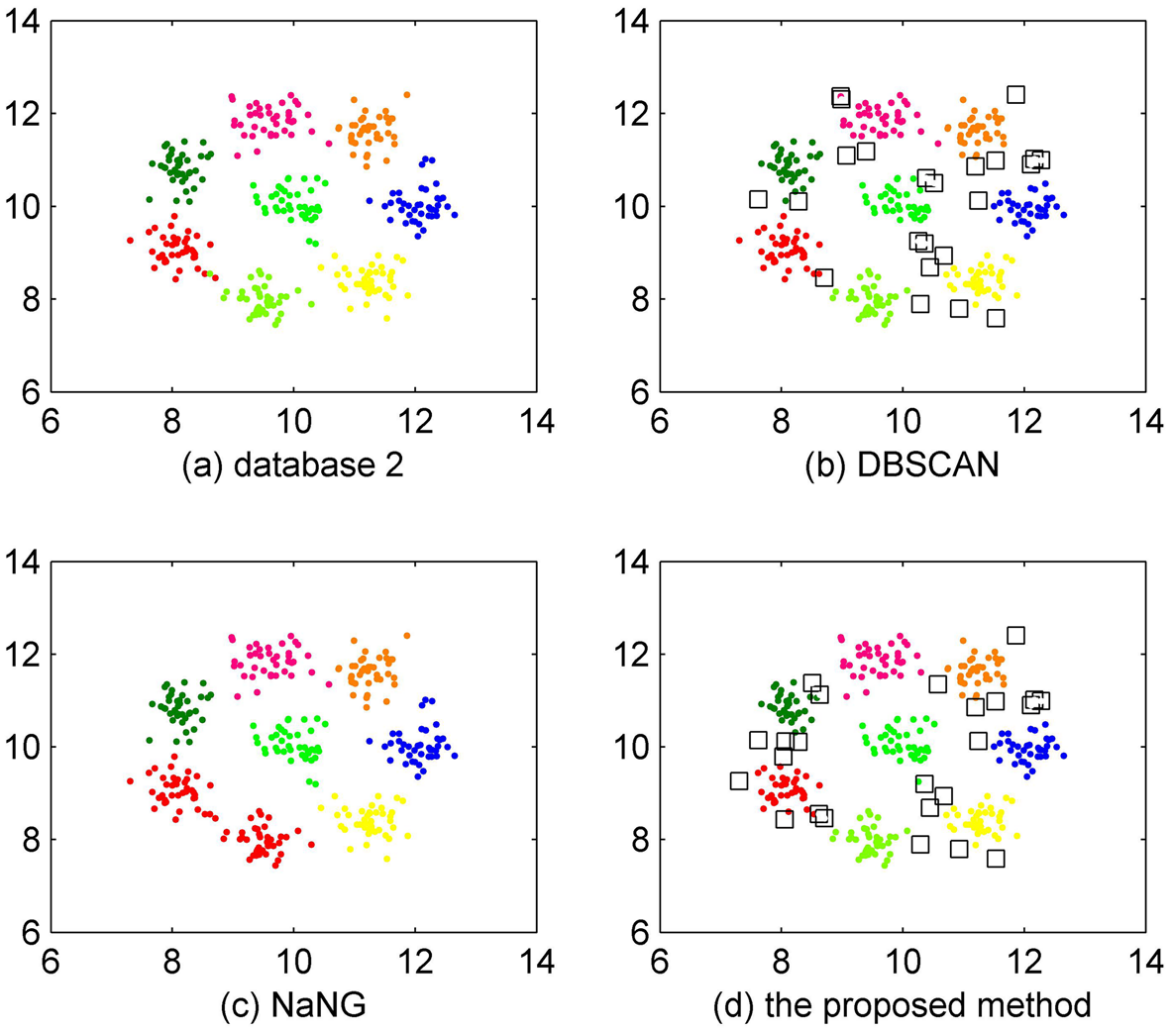
Clustering results of database 2.

The experimental results on database 2 are shown in Fig. 4. As shown in Fig. 4a, database 2 includes 320 objects and 8 clusters. As shown in Fig. 4b, the result of DBSCAN is tolerable with an accuracy of 92.5%. From the result shown in Fig. 4c, we can see that NaNG mistakenly combines two clusters into one. The accuracy of NaNG is 87.5%. As shown in Fig. 4d, the performance of the proposed method is the same as DBSCAN with an accuracy of 92.5%.

## Conclusion

Inspired by the mutual neighbor method and quantum computing, in this work, we present a quantum MMNG-based DBSCAN. Compared to the original DBSCAN, the proposed method performs better on databases with different local-density clusters. Furthermore, the proposed method is dramatically faster than its classical counterpart.
